# Expression and clinical significance of CMTM1 in hepatocellular carcinoma

**DOI:** 10.1515/med-2021-0221

**Published:** 2021-01-28

**Authors:** Xin Song, Shidong Zhang, Run Tian, Chuanjun Zheng, Yuge Xu, Tianxian Wang, Chunhua Bei, Huixia Zhang, Xiao He, Xiaonian Zhu, Shengkui Tan

**Affiliations:** Department of Epidemiology and Health Statistics, Guilin Medical University, Guilin, Guangxi, China

**Keywords:** CMTM1, HCC, TNM stage, prognosis

## Abstract

**Background:**

CKLF Like Marvel Transmembrane Domain Containing 1 (CMTM1) plays a role in breast cancer and lung cancer, but studies on the occurrence and development of CMTM1 in hepatocellular carcinoma (HCC) have not been reported.

**Methods:**

The Cancer Genome Atlas (TCGA) database and immunohistochemistry (IHC) were used to detect CMTM1 expression in HCC tissues. The relationship between CMTM1 expression and the clinicopathological characteristics of HCC patients was analyzed by chi-square test, and the relationship between CMTM1 expression and the prognosis of HCC patients was tested by the Kaplan–Meier model.

**Results:**

Bioinformatics analysis showed that the mRNA expression of CMTM1 was upregulated in HCC tissues, and low expression of CMTM1 is associated with longer disease-free survival in patients with HCC. Similarly, the survival time of HCC patients in CMTM1 high expression group was significantly shorter than that in CMTM1 low expression group. IHC detection indicated that CMTM1 protein was highly expressed in both HCC and adjacent non-tumor tissues, with a positive expression in 84% (63/75) of HCC tissues and 89.3% (67/75) of adjacent non-tumor tissues. Moreover, CMTM1 expression was related to family history and TNM stage of HCC patients (*P* < 0.05), but had no relationship with other clinicopathological characteristics. The survival analysis based on IHC results showed that the prognosis of HCC patients in CMTM1 negative group was significantly poorer than that in CMTM1 positive group (*P* < 0.05).

**Conclusion:**

CMTM1 has a high expression in HCC tissues and is related to the prognosis of HCC patients.

## 1 Introduction

Hepatocellular carcinoma (HCC), a common malignancy of the digestive system, is highly prevalent in sub-Saharan Africa and Asia [1]. According to recent statistics, HCC has the second-highest incidence and mortality in all cancers of China. More than 7,48,300 HCC cases are newly diagnosed globally each year, with China accounting for about 55% [2]. Most HCC patients are already in the middle and advanced stages when they are diagnosed. With the improvement of the medical level of surgery, comprehensive treatment based on clinical surgery significantly improves the therapeutic efficacy of HCC. However, the clinical cure rate of HCC and the long-term survival rate of patients continue to be low [3]. Furthermore, 60–70% of HCC patients have metastasis or recurrence within five years after tumor resection [4]. The occurrence and development of HCC are a complex process, including environmental and genetic factors. Although the carcinogenic mechanism of HCC has been extensively studied, the exact molecular mechanism of HCC has not been well-elucidated.

The chemokine-like factor superfamily (CKLFSF) is a new gene family first reported in 2001 [5]. It has been found that CMTM family members are extensively involved in tumor development, with CMTM2, CMTM3, CMTM4, CMTM5, and CMTM7 abnormally expressed in HCC tissues and associated with the survival time of HCC patients, which are important factors that affect the prognosis of HCC patients [6,7,8,9,10]. CMTM3 was identified to regulate the migration and invasion of gastric cancer cells and thought to be a clinical candidate marker for determining the prognosis of gastric cancer [11]. CMTM4 and CMTM6 can participate in immune escape through the synergistic protective effect of PD-L1 and are extensively involved in the process of tumor proliferation and metastasis [12,13]. CMTM5-v1 is found to inhibit prostate cancer (PCa) cells through the EGFR signaling pathway, and the loss of CMTM5 may be involved in the occurrence and development of PCa caused by deregulated EGFR. Meanwhile, CMTM5 may be associated with the inhibitory effect of tyrosine kinase inhibitors (TKIs) with EGFR and human epidermal growth factor-2 (HER2) activation [14]. Our previous studies have also found that CMTM4 and CMTM6 play a very important role in the occurrence and development of HCC [6,15].

CMTM1, a critical member of the CMTM family, is located on chromosome 16q22 and consists of 7 exons and 6 introns. According to the available studies, CMTM1 is highly expressed in testicular and tumor tissues, suggesting that CMTM1 might play a vital role in tumorigenesis [16,17]. Previous studies have found that CMTM1 is closely related to the occurrence, development, and treatment of breast cancer and lung cancer [16,18], but studies on the occurrence and development of CMTM1 in HCC have not been reported.

In this study, the expression of CMTM1 in HCC and adjacent non-tumor tissues was detected by immunohistochemistry (IHC) method. And then, we analyzed its relationship with clinicopathological features and prognosis of HCC patients. We aimed to provide a potential molecular marker for early diagnosis and therapy of HCC.

## 2 Materials and methods

### 2.1 Tissue samples

All research subjects were diagnosed with HCC by histopathology in the First Affiliated Hospital of Guilin Medical University from 2007 to 2015. Paired HCC and adjacent non-tumor tissues were obtained from 75 HCC patients through tumor resection. All patients had not received any type of treatment before surgery and had complete clinical data shown in Table 2. All patients were followed up by outpatient review or telephone. Survival time was calculated in months from the first day after surgery until the patient developed tumor metastasis, recurrence, death, or reached the end of follow-up. This study was approved by the ethics committee of Guilin Medical University (GLMC2014003) and obtained the written informed consent of each patient in compliance with the Helsinki Declaration.

### 2.2 Bioinformatics analysis

HCC gene expression profile files were downloaded from The Cancer Genome Atlas (TCGA) database including 423 CMTM1 expression profile samples (373 HCC specimens, 50 paracancerous specimens). Then, the data were collated by the R (3.6.1) package and followed by statistical analysis.

### 2.3 Immunohistochemistry (IHC)

Immunohistochemistry (IHC) was used to detect the protein expression of CMTM1. The specific steps are as follows: First, the tissue microarray with a thickness of 2 microns was baked in a 60℃ ovens for 2 h and dewaxed by xylene and hydrated with gradient ethanol after taking it out. Putting the tissue microarray in EDTA buffer (pH = 8.0), and repaired the antigen by high pressure and heating for 2.5 min. The tissue microarray was dripped with endogenous peroxidase blocker, incubated for 10 min to remove endogenous peroxidase, washed with PBS buffer, and then added goat serum to seal for 20 min. After drying, CMTM1 primary antibody (Abcam, Cambridge, MA, USA) was added and incubated overnight at 4℃. On the second day, after washing with PBS buffer solution, goat anti-rabbit secondary antibody was hereby added and incubated at room temperature for 30 min. After cleaning with PBS buffer, 3,3′-diaminobenzidine tetrahydrochloride (DAB) was given to treat the tissues to develop color and observed under a microscope for 5–10 min until the color development was appropriate. Finally, tissues were counterstained with hematoxylin, differentiated with 1% hydrochloric acid alcohol, rinsed back to blue with running water, dehydrated with gradient alcohol, transparentized with xylene, and fixed with neutral gum sealing tablets, the whole process without drying section conditions. All immunostained sections were evaluated blindly without knowing the clinicopathological information.

### 2.4 Assessment of IHC results

The assessment of CMTM1 expression in tissues was as follows: (1) five different fields of view were randomly selected at 400× microscopy for analysis. The scores for the percentage of positively stained cells were 0 for ≤5%, 1 for 6–25%, 2 for 26–50%, 3 for 51–75%, and 4 for >75%, respectively. (2) And the scores based on the intensity of staining were 0 for uncolored, 1 for light yellow, 2 for brown, and 3 for yellow-brown. Finally, the two scores were multiplied together: 0 was (−), 1–4 was (+), 5–8 was (++), and 9–12 was (+++). In this group, the positive and negative expression of CMTM1 was defined as score >4 and ≤4, respectively.

### 2.5 Statistical analysis

All data were statistically processed by SPSS19.0, and the expression difference between HCC and adjacent non-tumor tissues was compared by chi-square test. The survival probability was estimated by Kaplan–Meier method, and the survival curve between the groups was tested by Log-rank test. The difference was considered statistically significant when *P* < 0.05.

## 3 Results

### 3.1 CMTM1 expression in HCC tissues

**Figure 1:**
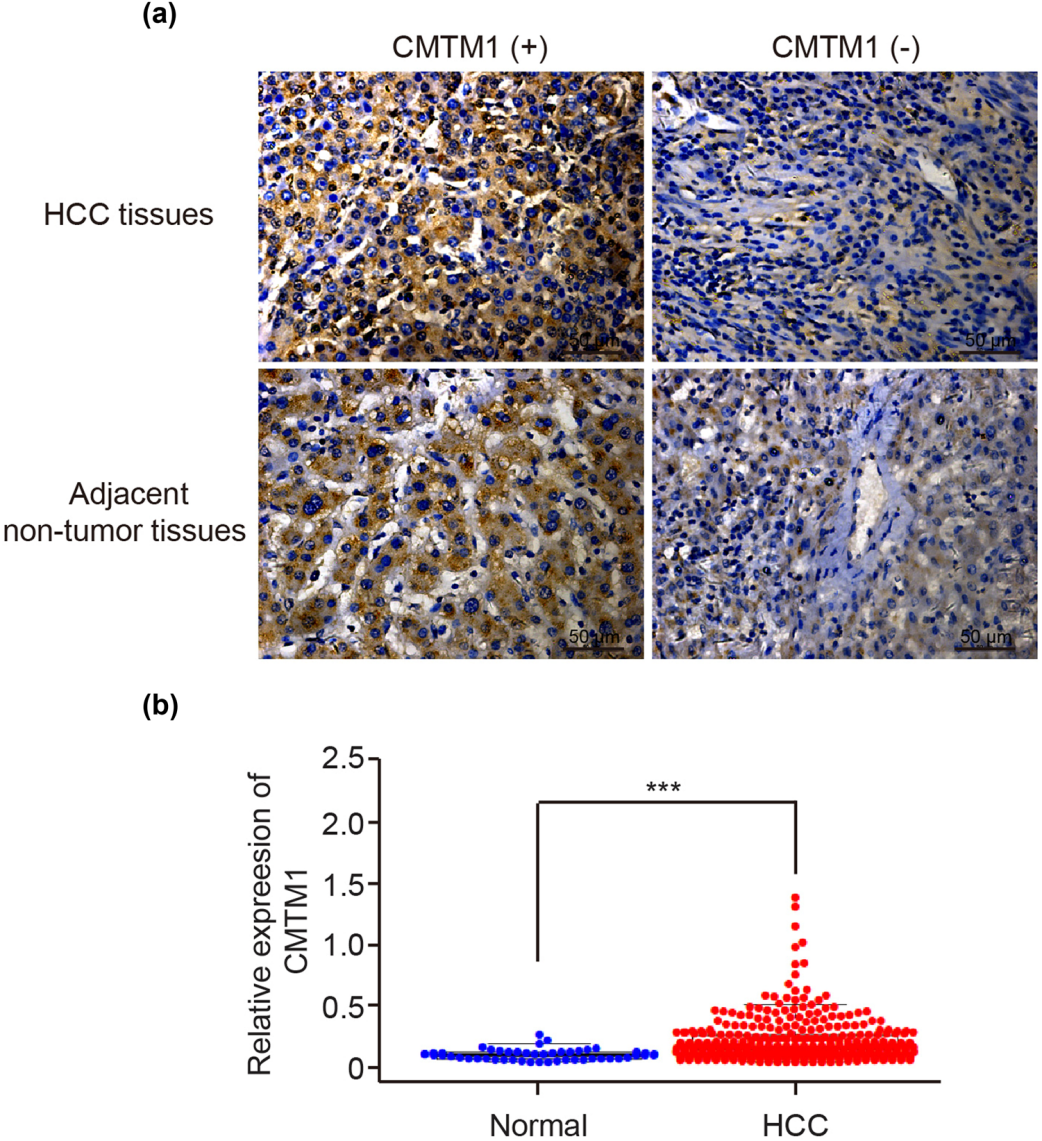
CMTM1 expression in HCC tissues. (a) Positive and negative expression of CMTM1 in HCC tissues and adjacent non-tumor tissues by IHC detection. (b) CMTM1 expression in normal liver and HCC tissues in the TCGA database. ^***^ *P* < 0.001.

We first conducted IHC to detect the protein expression of CMTM1 in HCC and paired adjacent non-tumor tissues. As shown in Figure 1a, the positive expression of CMTM1 in HCC and adjacent non-tumor tissues were 84% (63/75) and 89.3% (67/75), respectively, indicating a high expression of CMTM1 both in HCC and adjacent non-tumor tissues (Table 1, *P* = 0.079). And then we verified the expression of CMTM1 in HCC and normal liver tissues from the TCGA database. As shown in Figure 1b, we found that the mRNA expression of CMTM1 in HCC tissues was significantly higher than that in normal liver tissues (*P* < 0.05).

**Table 1:** CMTM1 expression in paired HCC tissues and adjacent non-tumor tissues

HCC tissues	Adjacent non-tumor tissues		Total
	Positive	Negative	
Positive	58	5	63
Negative	9	3	12
Total	67	8	75

Notes: *P* ＞ 0.05, *P* value is based on the McNemar *χ* ^2^ test. HCC: hepatocellular carcinoma.

### 3.2 Relationship between CMTM1 expression and clinicopathological characteristics of HCC patients

We further explored the relationship between CMTM1 expression and the clinicopathological factors of HCC patients based on IHC results. As shown in Table 2, CMTM1 expression was significantly associated with the family history and TNM stage of HCC patients (*P* < 0.05), while had no relationship with other clinicopathological features of HCC patients, such as gender, age, smoking, alcohol intake, HBV infection, liver cirrhosis, serum alpha-fetoprotein (AFP), tumor diameter, tumor number, or metastasis.

**Table 2:** Association between the expression of CMTM1 and clinicopathological features of HCC patients

Variables	Total	CMTM1 staining		*χ* ^2^	*P*
		Positive	Negative		
**Gender**					
Male	61	52	9	0.002	0.964
Female	14	12	2		
**Age, year**					
<50	34	28	6	0.441	0.506
≥50	41	36	5		
**Smoking**					
No	42	37	5	0.582	0.446
Yes	33	27	6		
**Alcohol intake**					
No	35	31	4	0.550	0.458
Yes	40	33	7		
**HCC family history**					
No	63	56	7	3.977	**0.046**
Yes	12	8	4		
**HBV infection**					
No	15	14	1	0.959	0.327
Yes	60	50	10		
**Liver cirrhosis**					
No	37	30	7	1.055	0.304
Yes	38	34	4		
**AFP (ng/mL)**					
<400	32	27	5	0.041	0.840
≥400	43	37	6		
**Tumor diameter (cm)**					
<5	39	31	8	2.219	0.136
≥5	36	33	3		
**Tumor number**					
1	40	34	6	0.008	0.930
≥2	35	30	5		
**Tumor grade**					
I + II	42	35	7	0.305	0.581
III + IV	33	29	4		
**TNM stage**					
T1 + T2	39	30	9	4.592	**0.032**
T3 + T4	36	34	5		
**Metastasis**					
No	48	41	7	0.001	0.987
Yes	27	23	4		

Notes: Bold values indicate significance.

### 3.3 Relationship between CMTM1 expression and HCC prognosis

**Figure 2:**
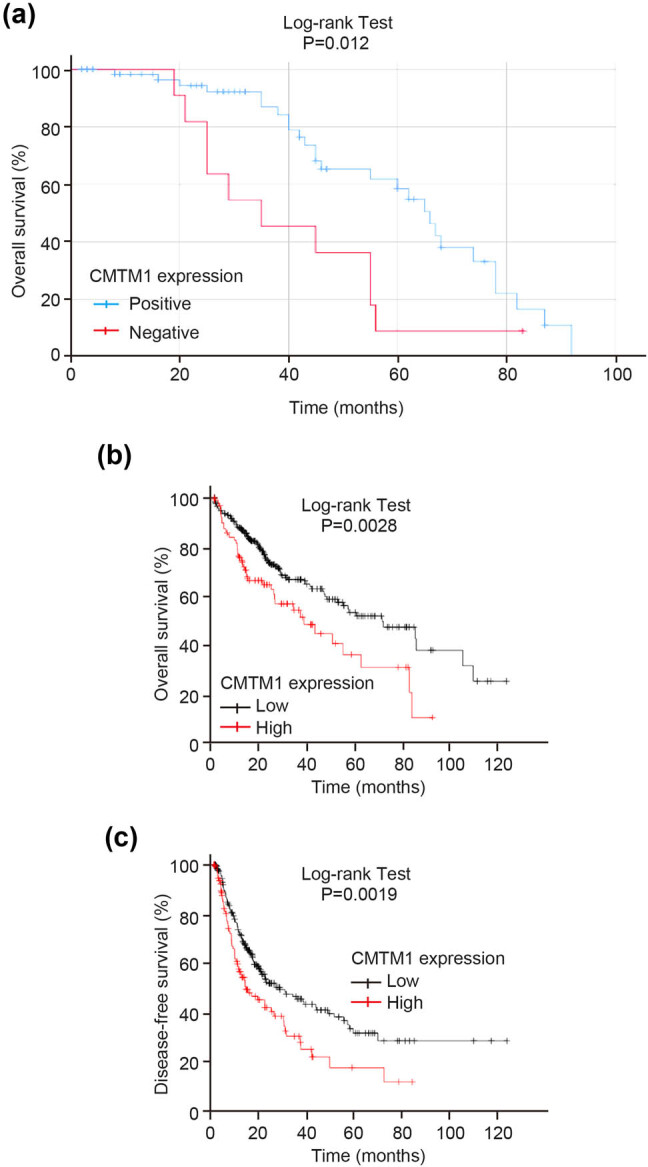
Relationship between CMTM1 expression and prognosis of HCC patients. (a) Analysis of the effect of CMTM1 expression on postoperative survival time of HCC patients using the Kaplan–Meier model based on IHC results. (b) Survival analysis of CMTM1 expression on postoperative survival time of HCC patients from the TCGA database. (c) Survival analysis of CMTM1 expression on disease-free survival time of HCC patients from the TCGA database.

To investigate the prognostic value of CMTM1 in HCC, we used the Kaplan–Meier model to analyze the effect of CMTM1 expression on the prognosis of HCC patients. And we found that the survival of HCC patients with CMTM1 positive expression was significantly higher than those with CMTM1 negative expression, as shown in Figure 2a. From the analysis of the TCGA database, the low expression of CMTM1 is associated with longer disease-free survival in patients with HCC and the tumor postoperative survival time of the CMTM1 high expression group was significantly shorter than that in the CMTM1 low expression group, as shown in Figure 2b and c. Further, COX proportional risk model found that CMTM1 was an independent prognostic factor for HCC patients with an OR of 2.475 (*P* = 0.017, 95% CI = 1.179–5.194, Table 3).

**Table 3:** COX regression analysis for overall survival of HCC patients after surgery

Variables	*B*	Wald	*P*-value	OR^a^	95% CI	
					Lower	Upper
CMTM1	0.906	5.740	**0.017**	2.475	1.179	5.194

Notes: Bold values indicate significance. OR^a^ adjusted for age and gender.

## 4 Discussion

As a member of the CMTM family, CMTM1 not only has a broad-spectrum chemotactic activity, but also plays a significant biological role in hematopoietic, immune, cardiovascular, and male reproductive systems. In the study of autoimmune diseases, it has been found that CMTM1 may be involved in the occurrence and development of arthritis by interacting with the C–C chemokine receptor 4 (CCR4) [19]. In addition, CMTM1 is associated with the occurrence of rheumatic diseases [20]. CMTM1 has also been reported to be highly expressed in testis, localized in spermatogonial cells, and secreted into spermatogenic tubules to participate in male reproductive activities [17]. Moreover, CMTM1 is recently found to be associated with the development, progression, and metastasis of various malignant tumors.

CMTM1-v17, an RNA splicing form of CMTM1, is highly expressed in both normal prostate tissues and prostate cancer-originated cell lines. It is a new androgen receptor co-inhibitor and exerts its tumor suppressor function by recruiting histone deacetylases, indicating that the CMTM1 gene is a potential tumor suppressor gene [21]. In MDA-MB-231 breast cancer cell lines, overexpression of CMTM1 can promote the proliferation of breast cancer cells and resist apoptosis induced by tumor necrosis factor-α (TNF-α) [16]. Besides, CMTM1 also promotes the invasion and proliferation of glioblastoma cells. High expression of CMTM1 is significantly related to the shorter overall survival of patients with glioblastoma, suggesting that CMTM1 is a priority target for glioblastoma [22]. CMTM1 is overexpressed in lymphoma cells, and the addition of CMTM1 polypeptide *in vitro* can induce apoptosis of lymphoma cells. This may be due to the interaction between CMTM1 and the calcium-loving cyclin ligand (CAML), which negatively regulates the calcium response of the endoplasmic reticulum (ER) and induces the activation of mitochondrial caspases and the release of cytochrome c, which in turn leads to cell apoptosis [23].

In this study, the mRNA expression of CMTM1 was upregulated in HCC tissues analyzed by bioinformatics, and its high expression was associated with poor prognosis for HCC patients. However, we didn’t find a different protein expression of CMTM1 between HCC and paired adjacent non-tumor tissues from IHC results, which may be due to different tumor sources, different malignant degrees, or different detection methods. From the analysis of the TCGA database, the survival rate of the CMTM1 high expression group was significantly lower than that in the CMTM1 low expression group. This is also different from our IHC results, and it may be caused by the small sample size of this experiment. Further studies of CMTM1 in HCC need to be elucidated from bigger population sample.

Combined with the relevant clinicopathological data, the protein expression of CMTM1 was significantly correlated with the family history and TNM stage of HCC patients. Further analysis by Kaplan–Meier model showed that the survival of HCC patients in the CMTM1 negative group was significantly lower than that in the positive group, indicating that the negative expression of CMTM1 might be related to the poor prognosis of HCC patients, which was consistent with the results of previous studies on CMTM family in HCC. COX survival analysis showed that CMTM1 was an independent risk factor for the prognosis of HCC patients. These results indicate that the negative expression of CMTM1 is associated with poor prognosis of HCC patients.

## 5 Conclusion

In summary, this study reports the relationship between the expression of CMTM1 with the occurrence and prognosis of HCC, which proves that the low expression of CMTM1 might be a risk factor for the poor prognosis of HCC patients. CMTM1 is expected to be a new molecular marker for predicting the poor prognosis of HCC in the future. However, due to the small sample size of this study, patient-related information such as whether the patients have received drug treatment after surgery is not comprehensive enough, which might lead to information bias. Therefore, whether CMTM1 can be a single prognostic molecular marker still needs to be fully validated.
